# The sensitivity of the zebrafish embryo coiling assay for the detection of neurotoxicity by compounds with diverse modes of action

**DOI:** 10.1007/s11356-023-27662-2

**Published:** 2023-05-22

**Authors:** Rebecca von Hellfeld, Christoph Gade, Lisa Baumann, Marcel Leist, Thomas Braunbeck

**Affiliations:** 1 School of Biological Sciences, University of Aberdeen, 23 St Machar Drive, Aberdeen, AB24 3UK UK; 2 National Decommissioning Centre, Main Street, Ellon, AB41 6AA UK; 3 Aquatic Ecology and Toxicology, Centre for Organismal Studies, University of Heidelberg, Im Neuenheimer Feld 504, 69120 Heidelberg, Germany; 4 In Vitro Toxicology and Biomedicine, Department Inaugurated By the Doerenkamp-Zbinden Foundation, University of Konstanz, Universitätsstraße 10, 78464 Constance, Germany; 5 Faculty of Science, Environmental Health & Toxicology, Vrije Universiteit Amsterdam, De Boelelaan 1105, 1081 HV Amersterdam, Netherlands

**Keywords:** Locomotor assay, Spontaneous tail movement, *Danio rerio*, Behavior profiling, Developmental toxicity testing, Alternative test method

## Abstract

In the aim to determine neurotoxicity, new methods are being validated, including tests and test batteries comprising in vitro and in vivo approaches. Alternative test models such as the zebrafish (*Danio rerio*) embryo have received increasing attention, with minor modifications of the fish embryo toxicity test (FET; OECD TG 236) as a tool to assess behavioral endpoints related to neurotoxicity during early developmental stages. The spontaneous tail movement assay, also known as coiling assay, assesses the development of random movement into complex behavioral patterns and has proven sensitive to acetylcholine esterase inhibitors at sublethal concentrations. The present study explored the sensitivity of the assay to neurotoxicants with other modes of action (MoAs). Here, five compounds with diverse MoAs were tested at sublethal concentrations: acrylamide, carbaryl, hexachlorophene, ibuprofen, and rotenone. While carbaryl, hexachlorophene, and rotenone consistently induced severe behavioral alterations by ~ 30 h post fertilization (hpf), acrylamide and ibuprofen expressed time- and/or concentration-dependent effects. At 37–38 hpf, additional observations revealed behavioral changes during dark phases with a strict concentration-dependency. The study documented the applicability of the coiling assay to MoA-dependent behavioral alterations at sublethal concentrations, underlining its potential as a component of a neurotoxicity test battery.

**Supplementary Information:**

The online version contains supplementary material available at 10.1007/s11356-023-27662-2.

## Introduction

Chemical-induced effects on the development of the nervous system have received increasing attention over the past decades, owing to detrimental events such as the thalidomide incident (Vargesson 2015) and the Minamata Bay disaster (Kitamura et al. 2020). However, the potential adverse effects of compounds have only been determined for a small fraction of the chemicals currently produced thus far (Delp et al. 2018). Among these adverse effects, neurotoxicity (NT) describes adverse effects of a biological, chemical, or physical agent on the structure and/or function of mature nervous systems (Vorhees et al. 2021) e.g., dopamine inhibition or degradation of receptors (e.g., Delp et al. 2021). In contrast, developmental neurotoxicity (DNT) results from the inhibition or alteration of structures in the nervous system during its development (Delp et al. 2018) and effects may become apparent after the time point of exposure (Aschner et al. 2017), e.g., alterations of neuronal differentiation, cell migration, or synaptogenesis (Chang 1998).

Most approaches to assess (D)NT have focused on mammalian test systems, in accordance with various the Organization for Economic Co-operation and Development (OECD) test guidelines (TGs). (OECD 2007)(Makris et al. 2009; van Thriel et al. 2012; Smirnova et al. 2014; Schmidt et al. 2017)Such studies, however, cost up to $2 million per compound, and their sensitivity and applicability to human risk assessment have repeatedly been questioned (Bailey et al. 2014; Aschner et al. 2017; Fritsche 2017; Monticello et al. 2017; Clark and Steger-Hartmann 2018). Along with the call to reduce, refine and replace current animal testing procedures (3Rs principle by Russell and Burch (1959)), the shortcomings of existing test systems have stimulated a shift-of-focus to alternative test methods. This includes cell based DNT test batteries, as well as in silico modeling, and in vivo models such as the zebrafish (*Danio rerio*) embryo.(Strähle et al. 2012)Escher et al. 2022 During early development from fertilization until an age of 144 h post fertilization (hpf), when exogenous feeding begins, zebrafish embryos are not protected under current EU animal welfare legislation (EU 2010), and have, thus, been classified as an “alternative” test system (Embry et al. 2010; Halder et al. 2010; Strähle et al. 2012). Their transparent chorion allows for immediate continuous monitoring of embryonic development and facilitates easy insight into organogenesis (Langenberg et al. 2003), which proved very comparable with that of other vertebrates (Meyers 2018).

Concerning potential (apical) endpoints of neurotoxicity, the development of locomotor behavior can be studied (Brockerhoff et al. 1995; Chandrasekhar et al. 1997; Blader 2000; Claudio et al. 2000; Arslanova et al. 2010; Brocardo et al. 2012; Baiamonte et al. 2016; Zindler et al. 2019b, 2020b). In addition to a standardized set of morphological criteria for acute toxicity in the fish embryo acute toxicity (FET) test OECD TG 236 (OECD 2013), a whole suite of additional endpoints of ecotoxicological relevance can be made accessible by minor modifications of the protocol, which may cover teratogenicity (Brotzmann et al. 2019; von Hellfeld et al. 2020; Escher et al. 2022), endocrine disruption (e.g., Islinger et al. 2002; Wang et al. 2019c; Yao et al. 2020; Zhou et al. 2020), induction of biotransformation (e.g., Goldstone et al. 2010; Loerracher et al. 2020b, a; Loerracher and Braunbeck 2021), or neurotoxicity (e.g., Bolon et al. 2011; Kais et al. 2015, 2017; Stengel et al. 2018; Zindler et al. 2020a; Brotzmann et al. 2021; Wlodkowic et al. 2022; Kämmer et al. 2022).

Given the complexity of the brain, direct detection of (D)NT and identification of affected regions remains challenging (Heyer and Meredith 2017), which has led to a focus on behavioral studies, along with neuropathological or neurochemical analyses (Bolon et al. 2011; Wlodkowic et al. 2022). Behavior has proven to be a sensitive indicator of (D)NT in general (Vorhees et al. 2021) and brain malfunction in specific (Piersma et al. 2012; Foster 2014; Fisher et al. 2019). In zebrafish embryos, spontaneous tail movement (synonymous with “coiling”) provides a potential readout for (D)NT compounds, as it represents an early form of motor activity (Vliet et al. 2017; Zindler et al. 2019b, a). The most prominent processes of early motoneuronal development are caused by changes in innervation and circuitry of the young locomotor system (Drapeau et al. 2002). At ~ 17 hpf, rhythmic tail movements with a frequency of about 0.6 Hertz (Hz), caused by a simple spinal-cord-dependent neurocircuit, emerge (Brustein et al. 2003). This behavior peaks at 19 hpf, and until 27 hpf (Saint-Amant and Drapeau 1998). This is followed by coordinated coiling behavior linked to the glutamatergic Rohon-Beard-neurons in the tail and the trigeminal neurons on the head of the embryo (Brustein et al. 2003). This integrated coiling behavior can be observed in the absence of external stimuli between 27 and 36 hpf (Saint-Amant and Drapeau 1998). This faster and more vigorous coiling controlled by glutamatergic and glycinergic receptors has been found to be particularly susceptible to chemical disruption (Saint-Amant and Drapeau 1998; Ramlan et al. 2017), thus being of particular interest in early developmental-stage exposure experiments.

The development of locomotor behavior can be assayed in embryos up to 48 hpf (“coiling assay”), and it has been accepted that the assay can detect neurotoxic effects in zebrafish embryos (Selderslaghs et al. 2010, 2013; Ramlan et al. 2017; Vliet et al. 2017; Basnet et al. 2019; Zindler et al. 2019b). Existing coiling behavior studies lack standardization, with some protocols used dechorionated embryos (de Oliveira et al. 2021), while others employed altered light:dark cycles (Kokel et al. 2013) or examined significantly shorter periods of coiling (Selderslaghs et al. 2013). The recording of coiling incidents can be accomplished either manually (Chen et al. 2012; Abu Bakar et al. 2017; Wang et al. 2019a, b) or automatically (Ogungbemi et al. 2021; Zhang et al. 2021). Some studies rely on expensive third-party software (Zindler et al. 2019a; de Oliveira et al. 2021), while others use open-access software which require coding knowledge (González-Fraga et al. 2019), or the use of simpler programs (Kurnia et al. 2021). Past studies have also shown considerable variation in the experimental setup: In some protocols, exposure was initiated with fertilization (Velki et al. 2017; Zindler et al. 2019b, a), others only began exposure during the coiling development itself (Vliet et al. 2017).

To elucidate the potential suitability of the coiling assay to detecting neurotoxicants with different modes of action (MoAs), and the assay’s sensitivity to these different MoAs, a selection of chemicals (acrylamide, carbaryl, hexachlorophene, ibuprofen, and rotenone) were tested in the coiling assay as proposed by Zindler et al. (2019a) (exposure from fertilization 48 hpf). Moreover, additional statistical analyses were conducted to elucidate the potential suitability of changes in the light cycle as external stimuli of coiling. With this approach, the present study will contribute to improving the coiling assay as a testing method for DNT in diverse compounds using an alternative test system.

## Material and methods

### Selection of test compounds

To prove the suitability of the modified coiling assay described by Zindler et al. (2019a) for the assessment of neurotoxicity by drug-like compounds and pesticides, compounds with different MoAs, molecular and cellular targets, chemical behavior, and environmental relevance were selected: (1) carbaryl, an acetylcholine esterase (AChE) inhibitor (Schock et al. 2012); (2) hexachlorophene, an antimicrobial known to cause defects in the central nervous system of rats (Kimbrough 1971) most likely elicited by myelinopathy (Jokanovic 2009); (3) ibuprofen, affecting behavior in developing zebrafish by significant decrease in early locomotion and hatching (Xia et al. 2017); (4) rotenone, a mitochondrial complex I inhibitor frequently employed in, e.g., Parkinson’s disease studies (Le Couteur et al. 1999; Betarbet et al. 2000); and (5) the industrial reagent acrylamide as a positive control for neurotoxicity (Spencer and Schaumburg 1975; LoPachin and Gavin 2008), which inhibits presynaptic vesicle cycling (LoPachin and Gavin 2012), elicits proteomic and transcriptomic changes in the central nervous system, and induces depression-like behavior in zebrafish (Faria et al. 2018). Previous work had shown dimethyl sulfoxide (DMSO) to be a suitable solvent for zebrafish embryo behavioral studies, with final concentrations of up to 1% not significantly altering the behavior of the embryos (Zheng et al. 2021; de Oliveira et al. 2021).

### Chemicals and test concentrations

All compounds listed above were selected based on the observation of tremors before hatching elicited at sublethal concentrations in zebrafish embryos in the fish embryo acute toxicity (FET) test (details not shown). A detailed description of all other observed endpoints induced by exposure to the selected endpoints can be found in (von Hellfeld et al. (2020). Within EU-ToxRisk, test compounds were distributed by the Joint Research Centre (Ispra, Italy); and shipping and storage was in accordance with manufacturers’ instructions. Acrylamide and hexachlorophene were obtained from Sigma Aldrich (Deisenhofen, Germany); carbaryl from Carbosynth (Compton, UK), ibuprofen and rotenone were supplied by TCI (dichrom, Eschborn, Germany) and DMSO, used as solvent for some of the compounds, was purchased from Honeywell (Offenbach, Germany). All other chemicals were purchased at the highest purity available from Sigma Aldrich, unless stated otherwise.

Physicochemical properties of the test compounds as well as test concentrations are summarized in Table 1. The highest exposure concentration for the coiling assay was selected to be below the 10% lethal concentration (LC_10_; measured at 96 hpf) previously determined according in FET tests according to OECD TG 236 (von Hellfeld et al. 2020). In detail, test concentrations were established as follows: (1) the highest concentration was selected to be between the 50% effect concentration (EC_50_; measured at 96 hpf) and the LC_10_, ensuring that effects can be observed without lethality. (2) The two following concentrations follow the EC_50_ and EC_10_, respectively. (3) The lowest tested exposure concentration was set to always be well below the EC_10_. For compounds with published EC and LC data from the FET test in accordance with OECD TG 236 (OECD 2013), a range-finding FET was conducted to ensure accuracy of data. For compounds not tested in the FET, a full assessment with 3 replicates was conducted to establish the EC and LC values.

**Table 1:** Physicochemical properties and concentration ranges of compounds tested in the coiling assay

Compound	CAS nr	g/Mol	Water solubility^1^		Log K_ow_^1^	DMSO [%]	Test concentration in coiling assay			
Acrylamide	79–06-1	71.08	390 g/L	(25 °C)	− 0.67	0	0.1–2.1	mM	7.5–15	mg/L
Carbaryl	63–25-2	201.22	110 mg/L	(22 °C)	2.36	0.5	1.5–37.3	µM	0.3–7.5	mg/L
Hexachlorophene	70–30-4	406.90	140 mg/L	(25 °C)	7.54	0.1	1.0–49.2	nM	0.4–20	mg/L
Ibuprofen	15687–27-1	206.28	21 mg/L	(25 °C)	3.97	0.5	1.5–145.4	µM	0.3–30	mg/L
Rotenone	83–79-4	394.42	0.17 mg/L	(25 °C)	4.10	0.1	1.0–20.3	nM	0.4–8	mg/L

^1^National Center for Biotechnology Information – PubChem: https://pubchem.ncbi.nlm.nih.gov/

All test solutions were prepared freshly prior to the experiment, using standardized water according to the OECD TG 236 (OECD 2013). Stock solutions were stored at 4 °C during the experiment and transferred to − 20 °C thereafter. For compounds requiring the use of a solvent (DMSO), the lowest possible final DMSO concentration was used (0.1% for hexachlorophene and rotenone, 0.5% for carbaryl and ibuprofen, see Table 1). Nominal concentrations were used; given a renewal of test solutions every 24 h, changes due to biotransformation, evaporation and adsorption were considered minimal for this experimental setup.

### Fish maintenance and exposure

Adult wild-type “Westaquarium” strain zebrafish, kept in the facilities of the University of Heidelberg Aquatic Ecology and Toxicology Research Group (license number: 35–9185.64/BH), were utilized for the production of zebrafish eggs. The maintenance and conditions, as well as the egg collection procedure were conducted according to Lammer et al. (2009). For a description of exposure in the FET test, see von Hellfeld et al. (2020).

### Coiling assay

**Fig. 1:**
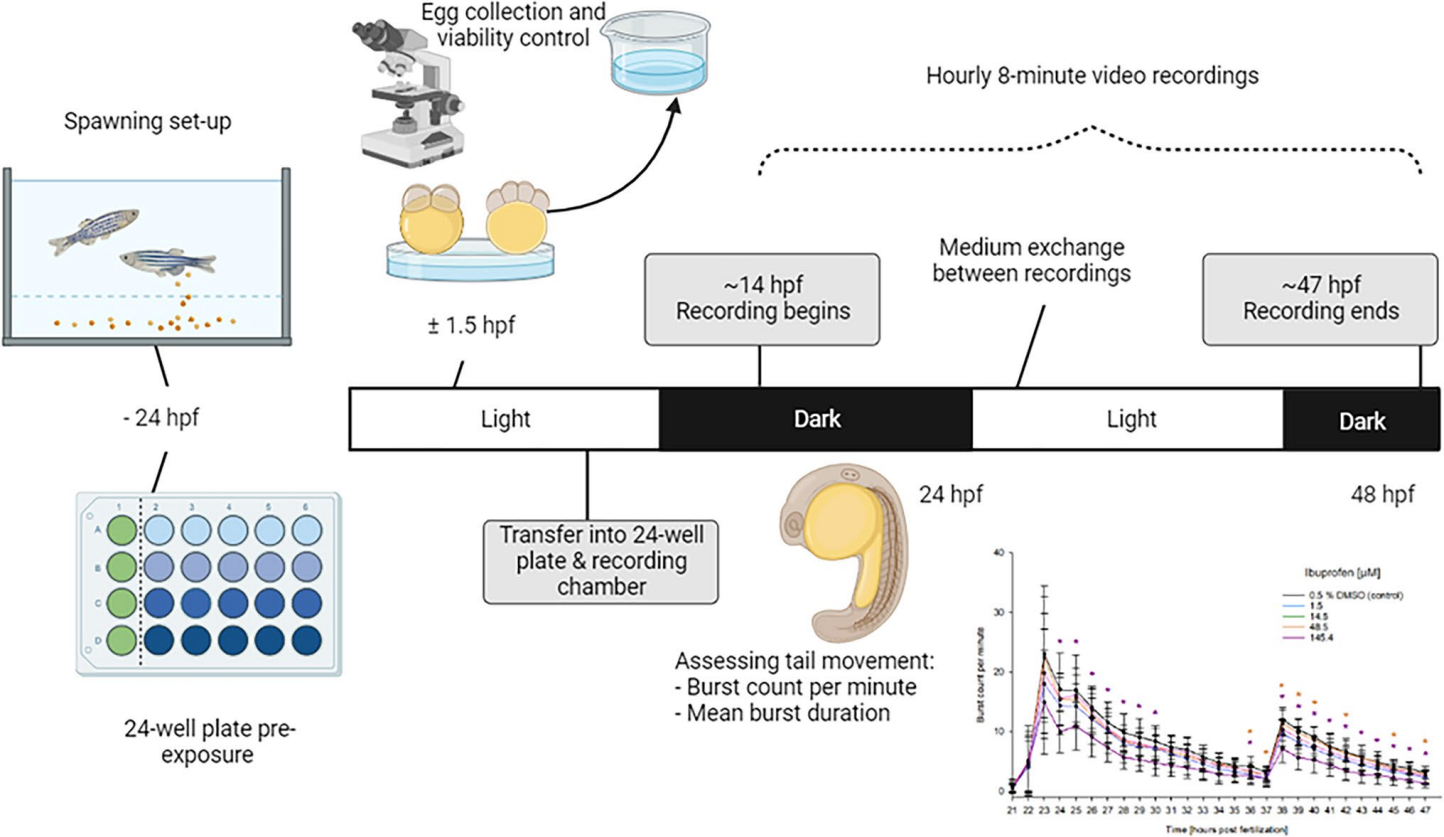
Setup and timeline of the coiling assay with zebrafish (*D. rerio*) embryos. A 24-well plate and the Teflon rings were pre-exposed to the respective test solutions for 24 h prior to exposure. Fertilized eggs were raised in crystallized glass dishes containing the respective exposure concentration or control medium until they were transferred into the 24-well plates (5 embryos per well, 20 embryos per concentration per replicate) and placed in the recording setup. Recording was performed at hourly intervals between 21 and 48 hpf, with two light regime changes (at 23.5 and 37.5 hpf). The test solutions were 100% renewed each day

The coiling assay (Fig. 1) was conducted in accordance with Zindler et al. (2019a). Fertilized eggs (< 2 hpf) were placed in 50 ml crystalizing dishes containing the test solutions (i.e., negative control/solvent control, or exposure concentrations are highlighted in Table 1) at 26.0 ± 1.0 °C and left in a HettCube 600R incubator (Hettich, Tuttlingen, Germany) for further development (*n* = 3 replicates: 20 embryos per concentration per replicate). At ~ 7 hpf, 5 embryos per treatment group were transferred to a pre-exposed 24-well plate and centered with a 5.3 mm-diameter polytetrafluoroethylene ring (ESSKA, Hamburg, Germany) at the bottom of the well. The test concentrations were randomly distributed on the plate to avoid instrumental bias due to proximity to heating elements of other interferences. The plate was placed on an acrylic glass lightbox (twelve infrared lights: 880 nm, 40° angle, 5 mm; Knightbright, Taiwan) in an incubator at 26.0 ± 1.0 °C with a 14/10 h light/dark regime. The incubator was set to switch off for 15 min every hour, 3 min prior to the onset of recording to avoid interference of the recordings with capacitor vibrations. Test solutions were renewed daily, replacing the plate in the incubator approx. 20 min before the next recording to allow for re-acclimatization. Hourly 8-min videos (mpeg-4, 25 frames/s) were recorded (camera: Basler acA1920–155 µm, Ahrensburg, Germany; lens: M7528-MP F2.8 f75mm, computar, Basler, Ahrensburg, Germany; filter: heliopan, RG850, Gräfelfing, Germany) utilizing the Ethovision™ Software (Noldus, Wageningen, Netherlands).

### Recording and management of coiling data

Videos were analyzed with the DanioScope™ Software (Version 1.1, Noldus, Wageningen, Netherlands). To prevent false positive/negative results, the software parameters were adjusted to the behavior of control group embryos (for details, see Supplementary Material 1). Data smoothing led to different *y*-axis scales in the graphs for mean burst duration for DMSO and the test compounds. Individuals moving too strongly could not be tracked efficiently by the software and were eliminated from tracking at the time point in question (for the percentage of individuals that could be tracked for the test compounds, see Supplementary Material 2).

Hourly recordings of 8 min were made of the 24-well plate, and the DanioScope™ software gave a mean value ± standard deviation of the measured behavioral parameters (“mean burst duration (seconds)” indicating the duration of continuous movement, and “mean burst count per minute,” i.e., the number of movements initiated each minute) per embryo for each recording event. Additionally, the response of the embryos to extinguishing the light at 37.5 h was examined, as it simulated an external stimulus. The so-called *step change* (SC) between recordings at 37 and 38 h was calculated by subtracting the 38 h mean value of the recorded parameter from the 37 h mean value. SC_10_ and SC_50_ values were calculated as the threshold for 10 and 50% deviation from controls.

### Data analysis and statistics

The DanioScope™ software was used to analyze the videos recorded and to convert them into mean values per individual per time point. From the data obtained, measurements provided for individuals who had to be omitted due to excessive movement were manually removed (for an example, see Supplementary Material 3).

Data analysis was conducted in a multi-step process: the data was first screened manually, and all individuals that had to be omitted from analysis (see above) were removed. The data was normalized to negative/solvent controls and analysis of variance (ANOVA)-on-ranks and Dunn’s *post-hoc* tests were conducted for each biological replicate using GraphPad Prism (v.6 for Windows; Statcon, Witzenhausen, Germany). The statistical analysis was conducted separately for each biological replicate and time point, since the fish used originated from different parent fish and since tests had to be run on different days, thus external influencing parameters could not be excluded. A deviation in behavior was an effect of exposure if at least two of three replicates found it to be statistically significant (*p* ≤ 0.05). All *p*-values are shown in Supplementary Material 4.

Mean values were normalized against controls, and the standard deviation was computed by taking the *n*th root of the sum of both standard deviations for *n* biological replicates. All graphs were created in SigmaPlot (v.14.0, Jandel-Systat, Erkrath, Germany), layouts were adjusted created in Inkscape (v.1.0.1, Free Software Foundation, Inc. Boston, USA).

## Results and discussion

### Methodological considerations

For the recording of coiling behavior, the following assay conditions proved to be optimal (variations not shown in detail): freshly fertilized zebrafish eggs were selected and exposed to the test solutions at latest 1.5 hpf. Each compound was tested at 4 concentrations well below LC_10_ values, with the lowest concentration selected to induce no effect. For quality assurance, untreated water and DMSO (where applicable) served as negative and solvent controls, respectively. Given that the camera was only capable of simultaneously capturing 5 columns on the 24-well plates, no internal positive control was tested.

Videos of 8 min were recorded every hour between 24 and 47 hpf. From these videos, the mean burst duration and mean burst count per minute were obtained as mean values per individual and time point. Here, burst refers to the movement initiated by the individual embryos. The burst duration refers to the length of time spent moving and the burst count per minute determines the amount of movement events initiated within an observed minute. Control group individuals (≤ 0.5% DMSO, as well as untreated) followed the previously described behavior development with two burst frequency peaks at ~ 24 hpf (Saint-Amant and Drapeau 1998) and 38 hpf. To account for biological variability, exposure groups were normalized to the corresponding control group obtained from the same zebrafish egg clutch. Differences between treatments and controls were considered to have been induced by exposure if an observation was made in at least two out of three biological replicates.

The coiling assay was conducted in accordance with the FET test (OECD 2013). Until approximately 72 hpf, developing zebrafish embryos are surrounded by the 1.5–2.5 µm thick acellular chorion, which consists of three layers pierced by pore canals (Hisaoka 1958; Laale 1977; Bonsignorio et al. 1996; Rawson et al. 2000) and has repeatedly been speculated to function as a barrier for the uptake of chemicals (Kais et al. 2013). The pores are evenly distributed over the chorion with diameters varying between 0.2 µm in unfertilized eggs (Hart and Donovan 1983) and 0.5–0.7 µm in fertilized eggs at the gastrula diameter (Rawson et al. 2000; Cheng et al. 2007; Lee et al. 2007). Between 24 and 48 hpf, the uptake of chemicals is limited to a molecular mass of 3 and 4 kilodalton, respectively (Pelka et al. 2017). Although all neurotoxicants tested are well below this critical molecular size (Table 1), exposure was extended to 120 hpf to prolong neurotoxicant exposure into life stages no longer protected by the chorion.

**Fig. 2:**
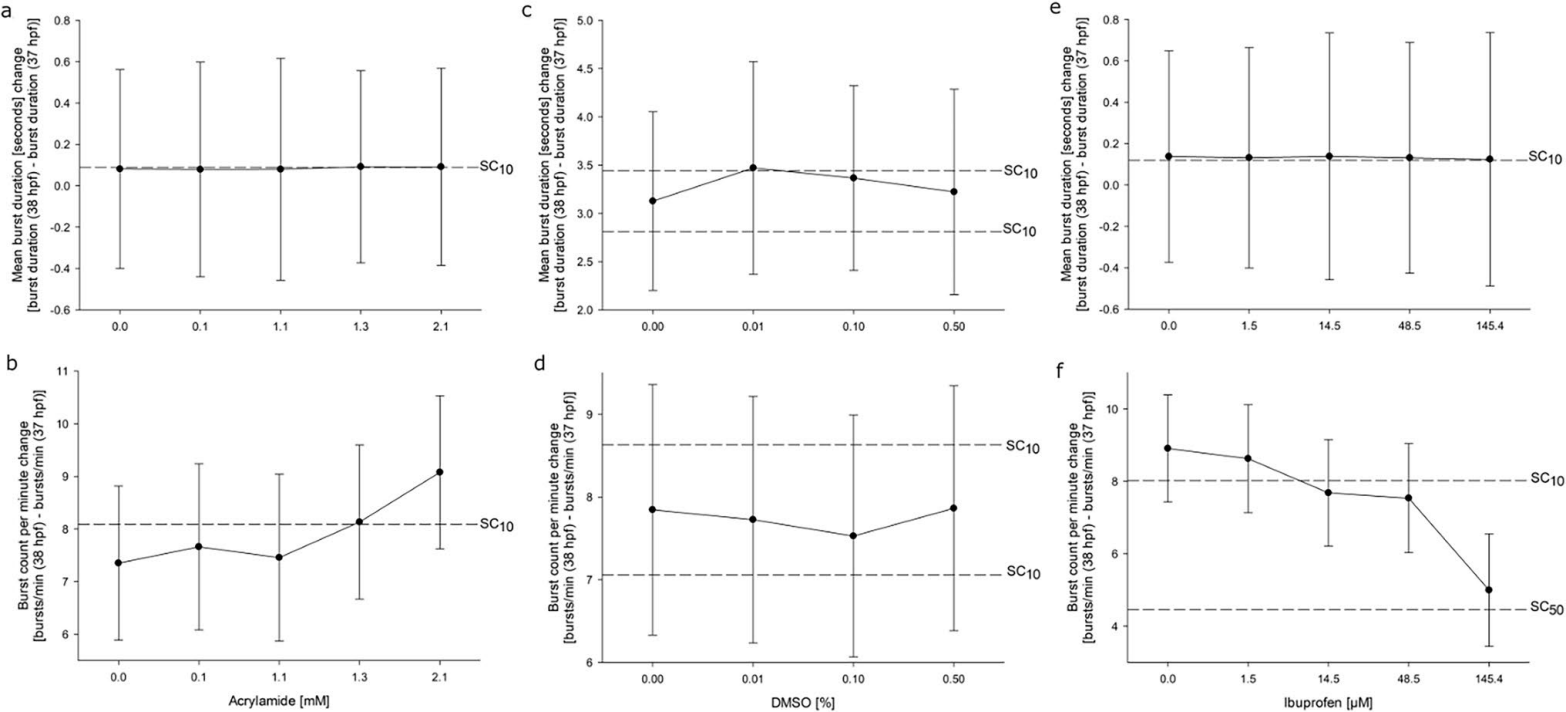
Effects of acrylamide, DMSO, and ibuprofen on the behavior of zebrafish (*D. rerio*) embryos during a change in the light regime. Graphs illustrate he difference in mean burst duration [seconds] (**a**, **c**, **e**) and burst count per minute (**b**, **e**, **f**) from 37 to 38 h old zebrafish embryos in the presence of acrylamide (**a**, **b**), DMSO (**c**, **d**), and ibuprofen (**e**, **f**). Data are given as the difference between the two time points ± SD from *n* = 3 replicates with 20 embryos per concentration/replicate. SC_10_ and SC_50_ indicate threshold for 10 and 50% deviation from the control groups, respectively

The addition of the step change analysis at the onset of the second dark phase (difference between 37 and 38 h; Fig. 2) allows for the examination of effects by external stimuli on behavior. In contrast to the minor changes induced by DMSO (Fig. 2 c, d), the step changes for both acrylamide (Fig. 2 a, b) and ibuprofen (Fig. 2 e, f) exceed 10% difference from controls (SC_10_), with ibuprofen almost reaching the 50% level (SC_50_). In contrast to acrylamide, which induces an increase in activity (Fig. 2 a, b), ibuprofen induces a decline (Fig. 2 e, f).

### Environmental relevance of neurotoxicant concentrations tested in the coiling assay

As summarized in Table 2, all neurotoxicants tested had a strong effect on coiling behavior in zebrafish embryos, with effects on burst count per minute usually being more pronounced than effects on burst duration. In fact, exposure to carbaryl, hexachlorophene and rotenone induced hyperactivity to an extent that required the exclusion of more than 30% of recorded embryos from the statistical analysis, making those videos unusable (Supplementary material 3).

**Table 2:** Summary of effects in the coiling assay with 21–47 h old zebrafish (*D. rerio*) embryos exposed to neurotoxicants

	Concentration	Mean burst duration (s)^1^	Age (h)	Light phase ^a^	Burst count per minute	Age (h)	Light phase ^a^	Surface water concentration ^b^
Acrylamide	1.3 mM	+ +	45	D2	+ + + + + +	42 43 44	D2	< 5 µg/L (70.34 µM)
	2.1 mM	+ +	45	D2	+ + + + +	38–40 41 45	D2	
Carbaryl	14.9 µM			L	– – – + +	24 27		Various rivers, Spain: 1.23–6.48 µg/ml (6.11–32.20 µM; Picó et al. (1994))
	29.8 µM			L	+ + +	24–27 28		
	37.3 µM	–	24	L	– – – – –	23 24	L	
	All	~	30–47	L, D2	~	30–47	L, D2	
Hexachlorophene	All	~	29–38	L	~	29–38	L	Urban drainage area (Greenboro, USA): 4.0–25.8 µg/L upstream (10–60 nM), 3.2–44.3 µg/L downstream (8–110 nM), and 15.2–48.3 µg/L in bottom water (40–120 nM)
Ibuprofen	48.6 µM	– –	30	L	– – – – – – – –	36–37 38–39 40–47	L D2 D2	USA (various samples): < 0.1 µg/L (0.5 nM)^b^
	145.4 µM	– – – – –	24 30–47	L D2	– – –	24–47	L, D2	USA (various samples): < 0.1 µg/L (0.5 nM); Various ground- and surface-water samples, UK: 2030 µg/L (126.77 µM; Spurgeon et al. (2022))
Rotenone	1.0 nM	– – –	22	D1	– – –	22	D1	
	10.1 nM	–	21				D1	Various ground- and surface-water samples, UK: 2030 µg/L (126.77 µM; Spurgeon et al. (2022))
	All	~	30–47	L, D2	~	30–47	L, D2	

^1^Activity: – minor reduction (*p*: 0.05–0.01); – – intermediate reduction (*p*: 0.01–0.001); – – – strong reduction (*p*: ≤ 0.001); + minor increase (*p*: 0.05–0.01); +  + intermediate increase (*p*: 0.01–0.001); +  +  + strong increase (*p*: ≤ 0.001); ~ hyperactivity that could not be statistically analysed

^a^Light phase: D1 – dark phase 1 until 13.5 h; L − light phase until 37.5 h; D2 − dark phase 2 until 47 h: ^b^PubChem (where available, locations provided)

Whereas effects by acrylamide and ibuprofen were only evident at concentrations higher than environmental levels, all other test compounds showed effects in the coiling assay well within the range of environmentally relevant concentrations. Concentrations of carbaryl in various rivers in Spain ranged from 6.11 to 32 µM (Picó et al. 1994), which is comparable to the range selected for the present coiling assays (1.5–37.3 µM). All concentrations tested for carbaryl induced severe hyperactivity, and concentrations ≥ 14.9 µM had an impact on the burst count per minute. Environmental concentrations of hexachlorophene in an urban drainage area in Greenboro (New York, USA) ranged from 8 to 120 nM (in up- and down-stream waters, and bottom water; Sims and Pfaender 1975), i.e., equivalent to or even higher than the concentrations tested in the coiling assays (1.0–49.5 nM), where hexachlorophene induced severe hyperactivity. Rotenone concentration in various ground- and surface-water samples in the UK were up to 127 µM (Spurgeon et al. 2022), which by far exceeded the concentration range of 1.0–20.3 nM tested positive in coiling assay.

### Effects of the solvent DMSO on the coiling behavior

Given the partly limited water solubility of the test compounds, DMSO was used as a co-solvent. DMSO has not only been shown to induce alterations at the molecular (protein) level during development at 0.01% (Turner et al. 2012) and to affect hatching and morphology (Chen et al. 2011) at < 1%, but has also been debated in the context of effects on developmental and behavioral endpoints (Maes et al. 2012; Turner et al. 2012), especially in light of a test system as sensitive as the coiling assay (Hallare et al. 2006). Whereas some more sensitive zebrafish strains expressed behavioral alterations after exposure to > 0.55% DMSO (Christou et al. 2020), wild-type zebrafish embryos did not show any effect on behavior at concentrations up to 1% DMSO in the coiling assay (de Oliveira et al. 2021).

**Fig. 3:**
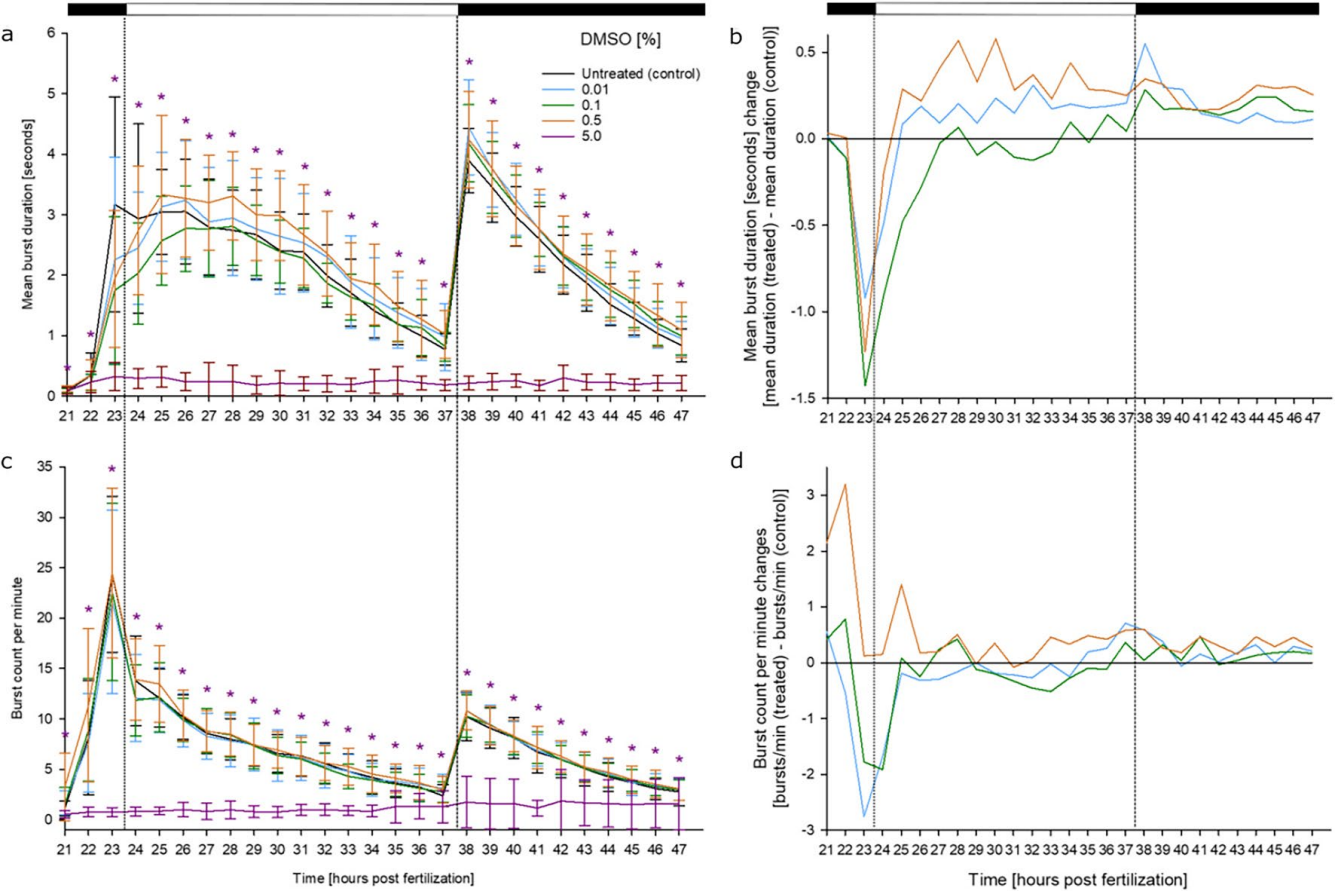
Effects of DMSO on spontaneous tail movement (coiling) of zebrafish (*D. rerio*) embryos during the light/dark cycles of the coiling assay: (**a**) Mean burst duration [seconds]; (**b**) normaized burst duration; (**c**)burst count per minute; (**d**) normalized burst count between 21 and 47 hpf of zebrafish embryos in the presence of various concentrations of DMSO (*n* = 3, 20 embryos per concentration/replicate). Normalized data were adjusted to negative controls (water) (**b, d**), and the 5% DMSO treatment group was excluded for better data visualization, highlighting the applicability of DMSO at ≤ 0.5 % concentration as a solvent in the coiling assay. **a**, **c:** Mean ± SD. Top bar: Light cycle pahse (black–dark; white–light). *: Time point and concentration (in corresponding color) of significant difference to controls (for statistical significance of changes over controls, see Supplementary Material 4)

Although the use of DMSO concentrations as low as 0.01% has generally been accepted for (eco)toxicological studies (OECD 2000; Jeram et al. 2005), a range of DMSO concentrations was also tested in the present study. Only at the highest test concentration of 5%, DMSO induced a significant inhibition of both mean burst duration and mean burst count per minute; in contrast, DMSO concentrations up to 0.5% did not produce any significant effect on zebrafish embryo coiling behavior (Fig. 3). Considering the response to the change in illumination at 37.5 h, only treatment with 0.01% DMSO induced an increase in the burst duration beyond the SC_10_ (Fig. 2 c, d). The large standard deviation of the observation, however, led to the assumption that this observation was due to biological variability. Overall, results thus confirm the suitability of DMSO as a solvent for behavioral studies, as already suggested by Chen et al. (2011) and Christou et al. (2020).

### Effects of acrylamide on the coiling behavior

**Fig. 4:**
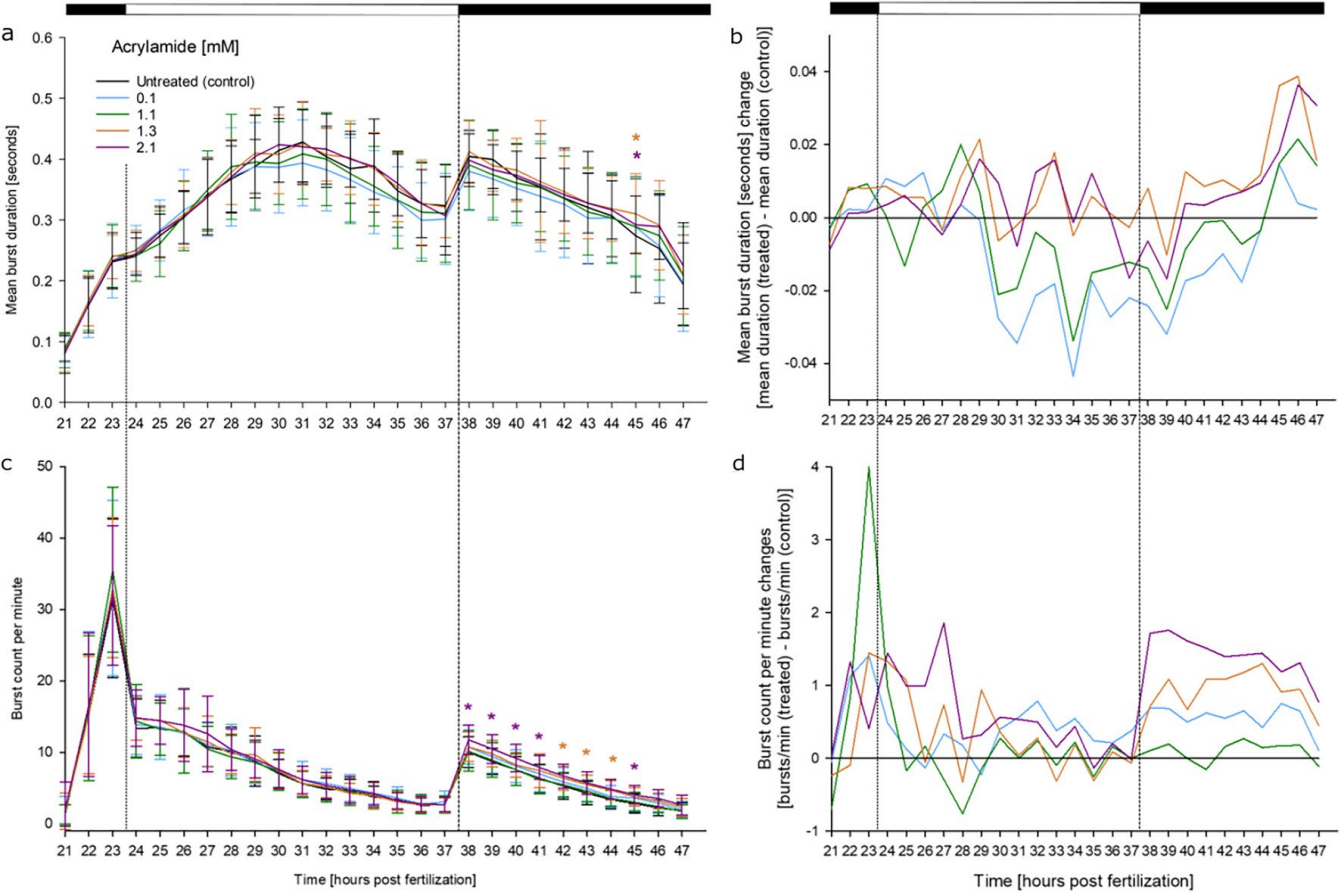
Effects of acrylamide on spontaneous tail movement (coiling) of zebrafish (*D. rerio*) embryos during the light/dark cycles of the coiling assay: (**a**) mean burst duration [seconds], (**b**) normalized burst duration, (**c**) burst count per minute, (**d**) normalized burst count between 21 and 47 hpf of zebrafish embryos in the presence of various concentrations of acrylamide (*n* = 3; 20 embryos per concentration/replicate). Acrylamide only significantly increased burst count per minute during the second dark phase (after 37.5 hpf) of the trial. **a**, **c** mean ± SD; **b**, **d** normalized to the untreated control group. Top bar: light cycle phases (black–dark; white–light). *: Time point and concentration (in corresponding color) of significant difference to controls (for statistical significance of changes over controls, see Supplementary Material 4)

In zebrafish embryos, acrylamide affected coiling in a concentration-dependent manner: exposure to acrylamide significantly increased burst counts per minute in the later stages of the coiling assay after the onset of the second dark phase, while the mean burst duration was unaffected (Fig. 4). In fact, acrylamide exposure increased the burst count per minute by more than 10% over controls already at the onset of the second dark phase, indicating that the increased activity in the later phases of recording were induced by the external stimulus of switching off the light.

In previous studies, acrylamide exposure was found to induce a “depression phenotype” concurrent with anxiety-like behavior in both embryos at 120 hpf and adult zebrafish (Prats et al. 2017; Faria et al. 2018, 2019). Inhibition of presynaptic vesicle cycling (LoPachin and Gavin 2012) resulted in reduced neurotransmitter release, membrane-reuptake, and vesicular storage, thus affecting, e.g., dopamine transport (LoPachin 2004; Barber and LoPachin 2004; LoPachin et al. 2006, 2007a, b; Barber et al. 2007). Since acrylamide did not affect axonal transport or protein synthesis, effects on presynaptic vesicle were concluded to be the direct toxic mechanism of acrylamide (LoPachin and Lehning 1994). Depression and anxiety-like behavior were also observed in rats, where acrylamide exposure decreased the monoamine neurotransmitters serotonin, norepinephrine, and dopamine (Dixit et al. 1981; Raushan et al. 1987), which could be linked to behavioral changes (Ruhé et al. 2007). Thus, the increase in the burst count per minute after the onset of the second dark phase observed in the present study might also be interpreted as an anxiety phenotype in response to the change in illumination.

### Effects of ibuprofen on the coiling behavior

**Fig. 5:**
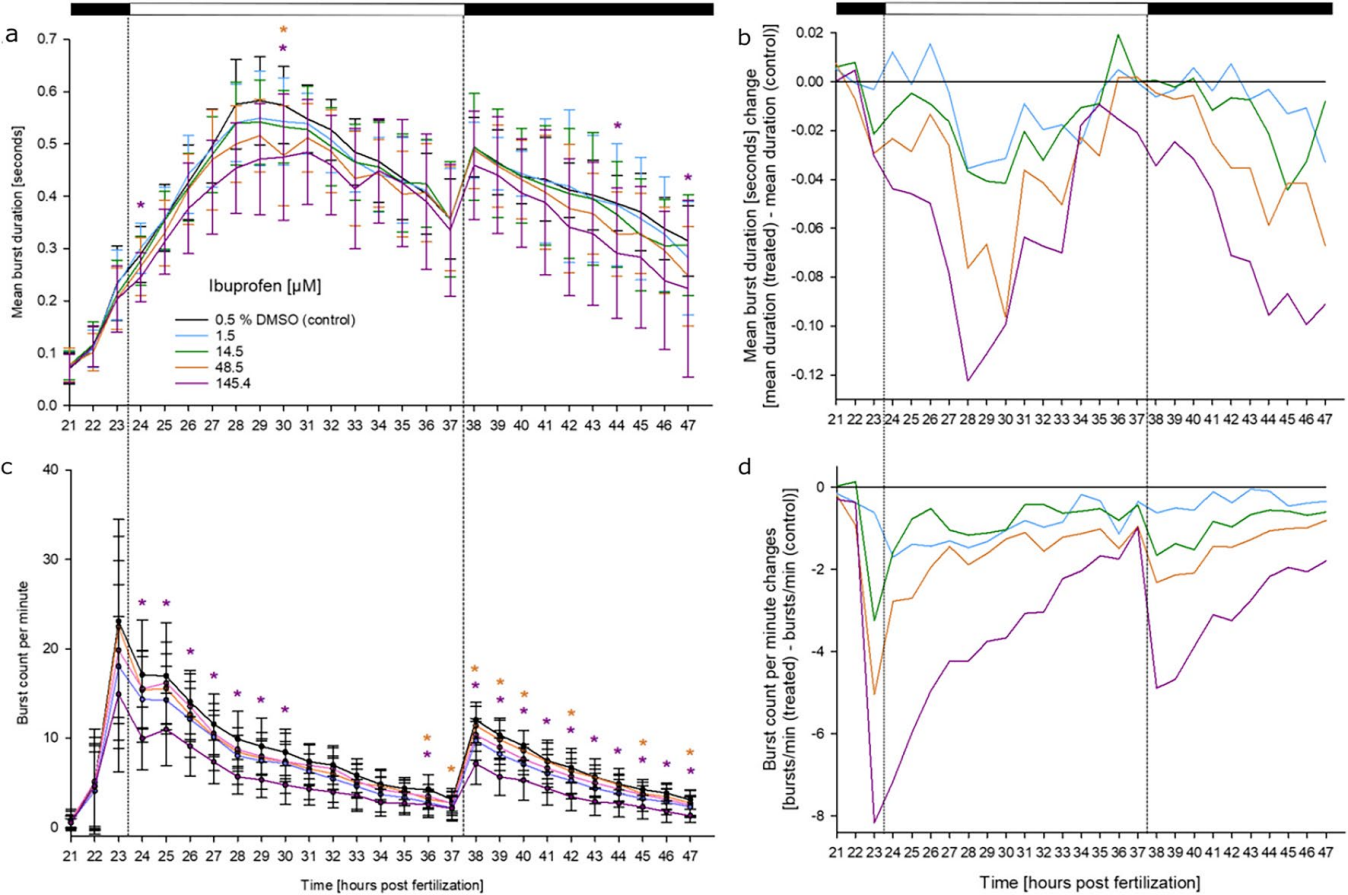
Effects of ibuprofen on spontaneous tail movement (coiling) of zebrafish (*D. rerio*) embryos during the light/dark cycles of the coiling assay: (**a**) mean burst duration [seconds], (**b**) normalized burst duration, (**c**) burst count per minute, (**d**) normalized burst count between 21 and 47 hpf of zebrafish embryos in the presence of various concentrations of ibuprofen (*n* = 3; 20 embryos per concentration/replicate). Ibuprofen exposure significantly reduced the burst count per minute during the entire assay in a concentration and time dependent manner. **a**, **c** mean ± SD; **b**, **d** normalized to the solvent control group. Top bar: light cycle phases (black–dark; white–light). *: Time point and concentration (in corresponding color) of significant difference to controls (for statistical significance of changes over controls, see Supplementary Material 4)

Exposure of zebrafish embryos to ibuprofen resulted in marked hypoactivity in both parameters, with burst counts per minute being more significantly attenuated than mean burst duration (Fig. 5). This observation was further supported by the step change analysis, where the mean burst duration was more strongly affected by the light change (Fig. 2): embryos exposed to 14.5 and 48.5 µM ibuprofen had a reduced burst count per minute below the SC_10_, while the 145.4 µM ibuprofen treatment group were close to SC_50_ levels. In this case, even the derived standard deviation of the observation was below the SC_10_.

Ibuprofen is known for its cyclooxygenase enzyme inhibition, reducing inflammation and pain by inhibiting prostaglandin and thromboxane formation (Bartoskova et al. 2013). Cyclooxygenases are necessary in early development of the embryo, as they regulate a vast number of hormones and developmental processes during early embryogenesis (Grosser et al. 2002). The reduction in burst counts per minute (and a trend towards reduction in burst duration) might thus be an indicator of impaired expression of glycine receptors, which are initially excitatory in zebrafish embryos (Brustein et al. 2013), before becoming inhibitory at around 30 h with the development of the potassium-chloride transporter 2 (Ben-Ari 2002; Brustein et al. 2013). A similar coiling phenotype was observed in the zebrafish mutant strain “shocked” (Cui 2005), where the under-expression of glycine receptors inhibited muscle fiber uncoupling and thus slowed down neurotransmission (Luna et al. 2004). A study conducted with the glycine receptor-blocker strychnine further induced an increase in multiple-coil events (with coiling continued for a longer duration), followed by longer recuperation phases (Cui 2005).

### Effects of carbaryl on the coiling behavior

**Fig. 6:**
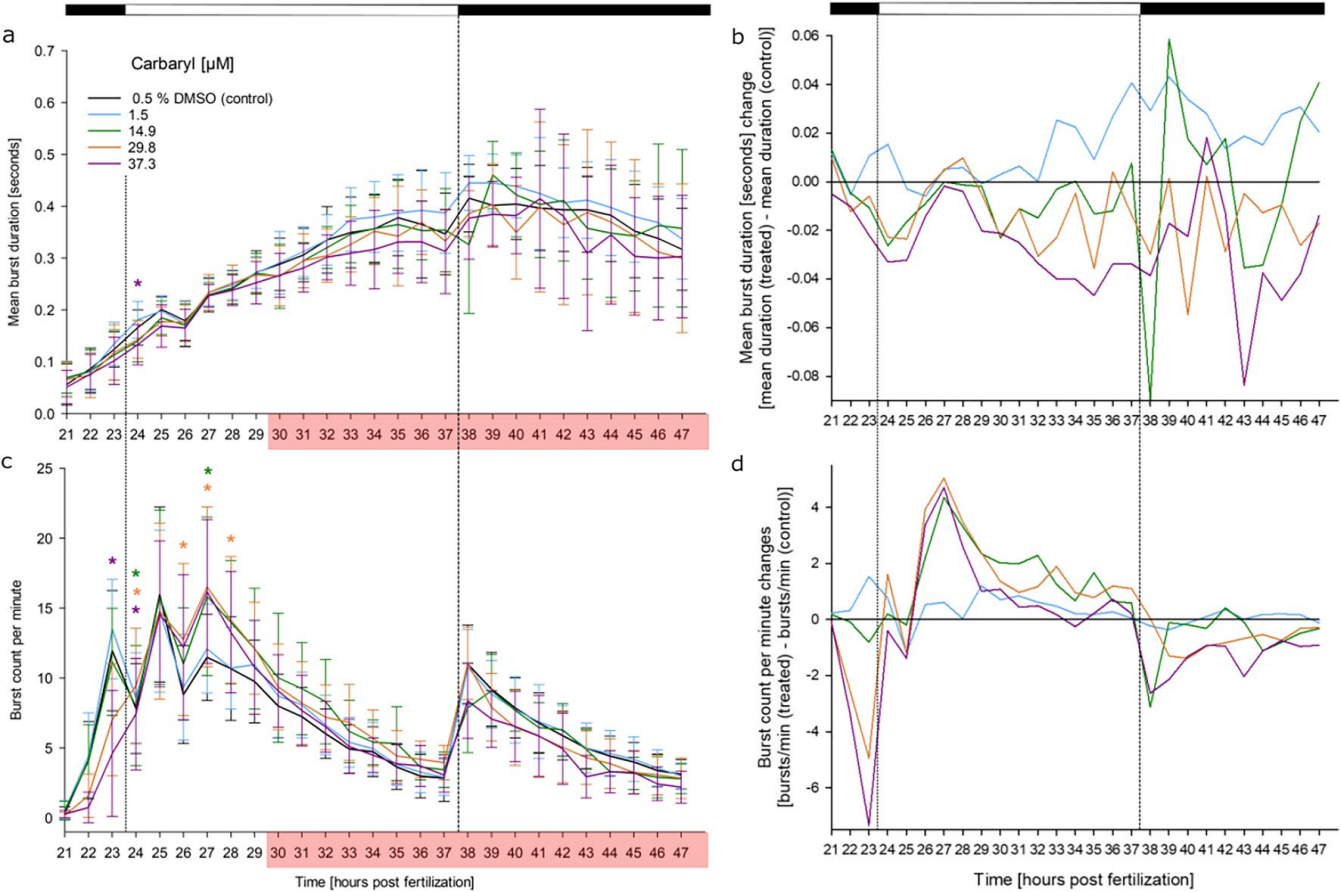
Effects of carbaryl on spontaneous tail movement (coiling) of zebrafish (*D. rerio*) embryos during the light/dark cycles of the coiling assay: (**a**) mean burst duration [seconds], (**b**) normalized burst duration, (**c**) burst count per minute, (**d**) normalized burst count between 21 and 47 hpf of zebrafish embryos in the presence of various concentrations of carbaryl (*n* = 3; 20 embryos per concentration/replicate). Carbaryl exposure significantly increased the burst count per minute in early stages of the assay before the onset of hyperactivity (red box) limiting statistical analysis. **a**, **c** mean ± SD; **b**, **d** normalized to the solvent control group. Top bar: light cycle phases (black–dark; white– light). *: Time point and concentration (in corresponding color) of significant difference to controls (for statistical significance of changes over controls, see Supplementary Material 4). Red box: at least 20% of organisms had to be excluded from the analysis of at least one of the exposure concentrations within this time frame

The analysis of effects by carbaryl, hexachlorophene and rotenone were more challenging, since a considerable number of individuals showed such a strong increase in activity that they had to be excluded from the analysis, since the program failed to track them with sufficient accuracy (see Supplementary Material 2). Yet, a statistically significant increase of burst counts per minute over DMSO controls could be seen from, e.g., 23 to 29 h for 14.9–37.3 µM carbaryl (Fig. 6). Due to the general hyperactivity at ≥ 30 hpf, the statistical trends could not be documented beyond 30 h.

The known acetylcholine esterase inhibitor carbaryl stimulates the activity of exposed organisms, e.g., mice (Andrieux et al. 2004), medaka (*Oryzias latipes*; Carlson et al. 1998) and zebrafish (Behra et al. 2002; Lin et al. 2007; Schock et al. 2012). Likewise, other acetylcholine esterase-inhibiting compounds like dichlorvos could also be determined as positive in the coiling assay (Zindler et al. 2019a). The hyperactivity in coiling observed in the present study can thus directly be linked to acetylcholine esterase inhibition as the underlying MoAs leading to reduced metabolization of acetylcholine and an overstimulation of neurons (Blacker et al. 2010).

### Effects of hexachlorophene on the coiling behavior

**Fig. 7:**
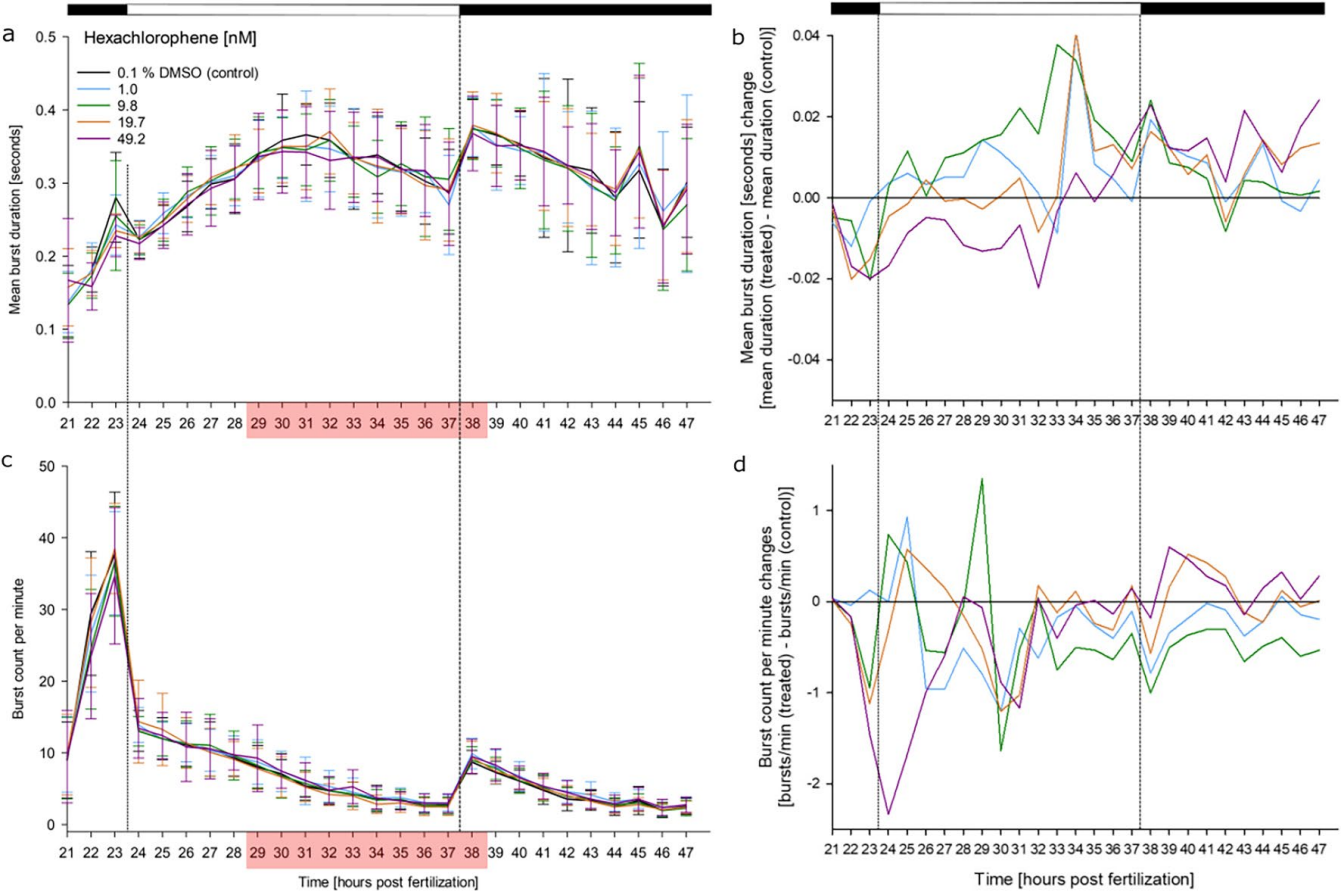
Effects of hexachlorophene on spontaneous tail movement (coiling) of zebrafish (*D. rerio*) embryos during the light/dark cycles of the coiling assay: (**a**) mean burst duration [seconds], (**b**) normalized burst duration, (**c**) burst count per minute, (**d**) normalized burst count between 21 and 47 hpf of zebrafish embryos in the presence of various concentrations of hexachlorophene (*n* = 3; 20 embryos per concentration/replicate). No statistically significant behavioral alteration was observed during hexachlorophene exposure, although hyperactivity (red box) was observed, which could not be analyzed. **a**, **c** mean ± SD; **b**, **d** normalized to the solvent control group. Top bar: light cycle phases (black–dark; white–light). Red box: at least 20 % of organisms had to be excluded from the analysis of at least one of the exposure concentrations within this time frame

Exposure of zebrafish embryos to hexachlorophene only induced temporary hyperactivity between 29 and 38 hpf; this trend, however, again failed to reach statistical significance due to the increase in general hyperactivity (Fig. 7).

In humans, accidental exposure to hexachlorophene caused central nervous system disruption and structural brain deformation (Powell et al. 1973; Martin-Bouyer et al. 1982; World Health Organization 2006). In mice and baboons, hexachlorophene exposure led to lethargy and reduced activity prior to lethality during episodes of convulsion (Tripier et al. 1981). Moreover, hexachlorophene has been implicated in disrupting the ion gradient across membranes, causing edema as well as demyelination (Jokanovic 2009). Such phenotypes of distal degeneration of some axons of both the peripheral and central nervous systems (polyneuropathy) could be associated with single or short-term exposure to various organophosphates (e.g., chlorpyrifos, dichlorvos, methamidophos, phosphamidon, and mevinphos) as well as certain carbamates, which initially lead to muscle cramps and spasms, before they induced progressive weakness and reduced reflexes (Lotti and Moretto 2005). Demyelination might, thus, also be speculated to be the underlying mechanism of the changes in behavioral parameters observed in the present coiling assays with hexachlorophene.

### Effects of rotenone on the coiling behavior

**Fig. 8:**
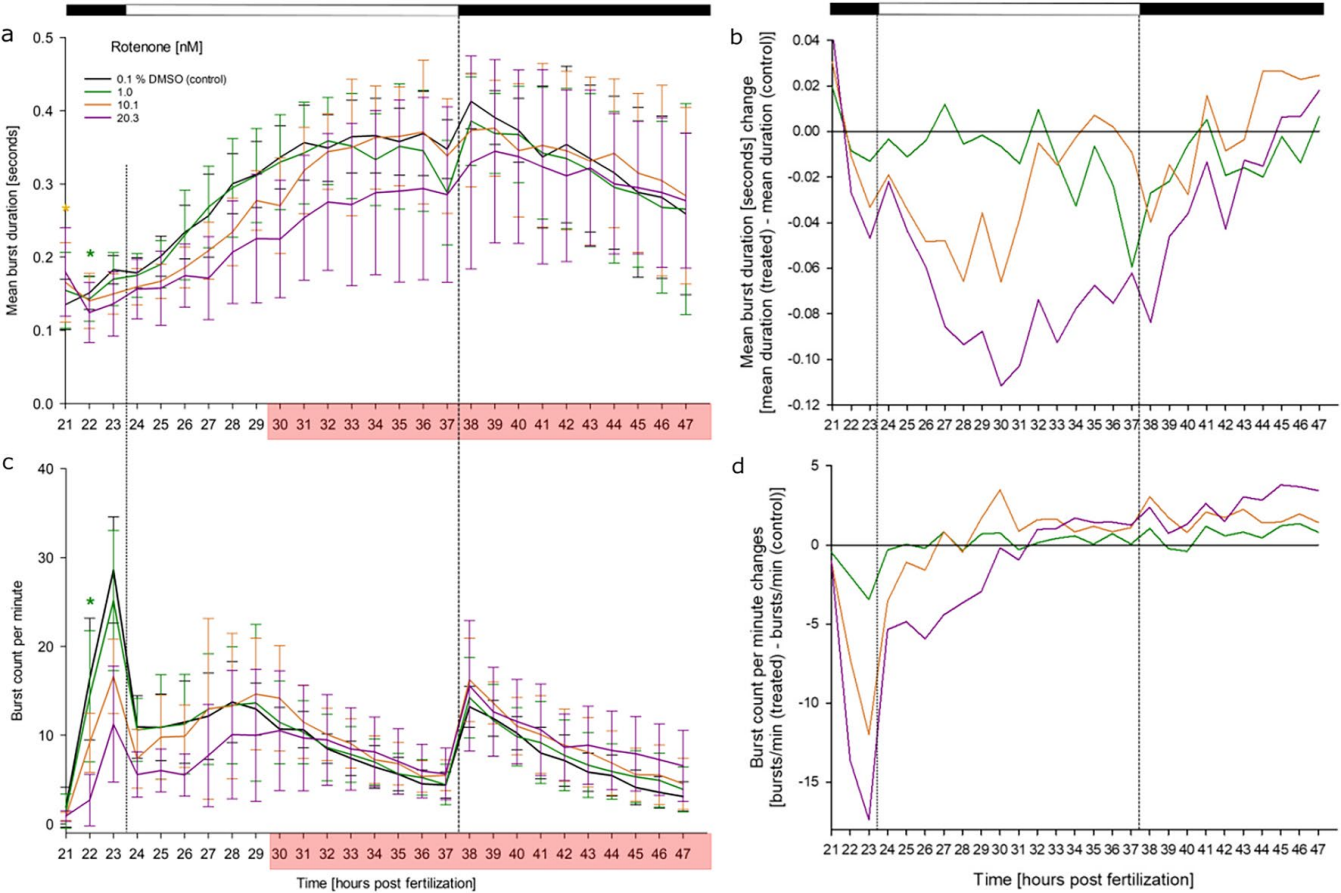
Effects of rotenone on spontaneous tail movement (coiling) of zebrafish (*D. rerio*) embryos during the light/dark cycles of the coiling assay: (**a**) mean burst duration [seconds], (**b**) normalized burst duration, (**c**) burst count per minute, (**d**) normalized burst count between 21 and 47 hpf of zebrafish embryos in the presence of various concentrations of rotenone (*n* = 3; 20 embryos per concentration/replicate). No statistically significant behavioral alteration was observed during rotenone exposure, although hyperactivity (red box) was observed, which could not be analyzed. **a**, **c** mean ± SD; **b**, **d** normalized to the solvent control group. Top bar: light cycle phases (black–dark; white–light). *: Time point and concentration (in corresponding color) of significant difference to controls (for statistical significance of changes over controls, see Supplementary Material 4). Red box: at least 20% of organisms had to be excluded from the analysis of at least one of the exposure concentrations within this time frame

Interestingly, rotenone exposure initially induced a reduction in both burst count per minute and mean burst duration, with some intermittent statistical significance. However, from 30 hpf extreme hyperactivity was observed, which prohibited conclusive statistical analysis (Fig. 8).

For research purposes, rotenone has frequently been used to induce motor and non-motor Parkinson’s disease via progressive loss of dopaminergic neurons in the substantia nigra (Le Couteur et al. 1999; Betarbet et al. 2000). As a mitochondrial complex I inhibitor, rotenone leads to enhanced mitochondrial reactive oxygen species production, impaired energy metabolism, proteasomal dysfunction, and finally apoptosis of dopamine neuronal cells (Li et al. 2003). The ability of rotenone to cross the blood–brain barrier is thought to play a vital role for its severe toxicity (Tanner et al. 2011).

In adult zebrafish, however, behavioral alterations, including decreased locomotor activity, were only seen in response to 1-methyl-4-phenyl-1,2,3,6-tetrahydropyridine (MPTP), but not with rotenone, where even systemic administration did not induce effects (Bretaud et al. 2004). In contrast, juvenile zebrafish showed loss of dopamine neurons in association with decreased locomotion and cardiac defects (Ünal et al. 2020; Wang et al. 2017). However, reports on the effects of rotenone on adult zebrafish are not consistent, since Wang et al., (2017) reported that rotenone-treated fish spent less time swimming at a fast speed, indicating a deficit in motor function. In a light–dark box test, rotenone-treated fish exhibited longer latencies to enter the dark compartment and spent more time in the light compartment, reflecting anxiety- and depression-like behavior. Furthermore, rotenone-treated fish showed less of an olfactory preference for amino acids, indicating olfactory dysfunction. Overall, behavioral alterations induced by rotenone exposure have been associated with decreased levels of dopamine in the brain (Wang et al. 2017).

### The coiling assay in the context of testing for (developmental) neurotoxicity

One in every six children has a developmental disability, and in most cases these disabilities affect the nervous system (Boyle et al. 1994). From the list of 80,000 chemicals registered for commercial use with the United States Environmental Protection Agency (US EPA) and 62,000 chemicals already in use when the Toxic Substances Control Act was enacted in the USA in 1977 (US EPA 1998), Grandjean and Landrigan (2006) identified 201 industrial chemicals as neurotoxic to humans, covering metals and inorganic compounds, organic solvents, numerous pesticides, and a multitude of other organic compounds. They argued that this evidence did by far not represent the true potential for industrial chemicals to cause neurodevelopmental disorders and concluded an urgent need for systematic testing for (D)NT. The need to identify (D)NT substances has, therefore, continued to grow with the continuous increase in the number of chemical compounds in human use, and a multitude of screening assays for (D)NT have been developed.

Various protocols have been developed for the coiling assay, which has been designed to identify effects on early behavior of zebrafish embryos through the assessment of spontaneous tail coiling (Selderslaghs et al. 2010, 2013; Velki et al. 2017; Wang et al. 2018; Zindler et al. 2019b, a; Bachour et al. 2020; Guo et al. 2021; Kurnia et al. 2021). Behavioral profiles become more complex, when exposure to neurotoxicants induces hyperactivity at low concentrations (e.g., through their ability to inhibit acetylcholine esterase) and hypoactivity at higher concentrations. This is the case for compounds which e.g., overstimulate the cholinergic system (Stehr et al. 2006; Küster and Altenburger 2007) or interact with γ-aminobutyric acid-gated chloride channels (Raftery and Volz 2015; Ogungbemi et al. 2019). Whereas many authors discussed the development of coiling behavior per se and the time patterns of movements, little distinctions have been made between the type of movements, which are more commonly grouped under the term “frequency” (Stehr et al. 2006; Tierney 2011; Velki et al. 2017). The present study, however, indicates that more attention should be given to a more in-depth analysis of behavioral patterns throughout the coiling period within fish development.

### Further considerations

This publication was designed as a pilot study, highlighting the potential of the coiling assay in detecting developmental neurotoxins with diverse MoAs. The presented data are based on five compounds only, and for method validation a larger database would be required. However, the data presented could provide guidance for future research, aiding the development of a standard operating procedure.

One limitation highlighted in this study is the issue of adequately analyzing severe hyperactivity using the program presented. Other studies have shown that, e.g., MATLAB can successfully track more extensive embryonic movements (González-Fraga et al. 2019). This, however, requires adequate coding skills, thus being of limited applicability for some. Although it may be possible to discern the movement manually in many cases, this might introduce observational bias, and thus, the comparability of results between different researchers could be impacted. At this point, there seems to be no viable solution to the highlighted analysis issue, other than potential future program updates or developments of novel analysis methods.

It should be noted that during all early development assays (including the FET test and the coiling assay), protocols highlight the necessity to follow a given light:dark cycle for natural development (e.g., OECD 2013; Zindler et al. 2019b; Braunbeck et al. 2020). Alterations of wavelength or duration of lighting have shown that rearing under conditions deviating from those applied in the present study led to reduce survival and hatching success, as well as increased developmental malformations (Villamizar et al. 2014). This can be explained by the development of the zebrafish eye, which begins at around 10 hpf and has differentiated into retinal ganglion cells and the optic nerve by ~ 28 hpf (Morris and Fadool 2005). The eye is structurally fully formed by 72 hpf (Glass and Dahm 2004), with a precise visual startle response detected at ~ 68 hpf (Easter Jr and Nicola 1996) and the light–dark response becoming evident not much later (Morris and Fadool 2005). A true optokinetic response, comparable to that observed in adult zebrafish becomes evident around 96 hpf but can initially be observed just after hatching (Easter and Nicola 1997). However, physical responses to changes in light conditions have been observed much earlier, in embryos during the coiling assay (e.g., Kokel et al. 2013; Zindler et al. 2019b). While the zebrafish embryo eye may thus not be fully developed or functioning, it can be assumed, that responses to large-scale visual stimuli such as light changes can already be perceived and lead to changes in behavior.

## Conclusions and perspectives

In the present study, a modified version of the coiling assay initially presented by Zindler et al. (2019a) was utilized to address some of the gaps in current protocols for the analysis of changes in zebrafish embryo behavior at sublethal concentrations. Additionally, this work has highlighted the suitability of the coiling assay as presented here to the detection of neurotoxic compounds with diverse MoAs. Moreover, the coiling assay holds the potential to determine compound- or MoA-specific behavioral profiles. However, while the assay was successful in determining behavioral alterations for all test compounds, excessive increases in locomotor behavior, albeit observable, proved to technically overstrain current tracking software systems. Nevertheless, the coiling assay holds great potential for the assessment of neurotoxic compounds, and would also be highly applicable in a test battery setting, as this would allow bringing, e.g., data from in vitro neurotoxicity assays like the neurite outgrowth impairment in human mature dopaminergic neurons (NeuriTox) assay (Delp et al. 2018) or the neurite outgrowth impairment in human iPSC-derived immature dorsal root ganglia neurons (PeriTox) assay (Hoelting et al. 2016) into a more functional context.

The software used in the present study allowed to read out several parameters for further analysis, and an in-depth analysis of effects by the set of neurotoxicants revealed that the test parameters are not addressed stereotypically by different compounds, suggesting the existence of MoA-specific effect profiles. The development of new and improved hard- and software allowing for a better analysis of individuals are likely to further comprehensive toxicity testing. Given the comparatively short experimental duration, the most time-consuming aspect of the coiling assay remains the software-based analysis of videos and subsequent data processing. If this step could be automated as suggested by González-Fraga et al. (2019), Ogungbemi et al. (2020, 2021) as well as Kurnia et al. (2021), the coiling assay with zebrafish embryos might even be developed into a high throughput alternative test method for neurotoxicity testing.

## Supplementary Information

Below is the link to the electronic supplementary material.

Supplementary file1 (DOCX 56 KB)

Supplementary file2 (PPTX 64231 KB)

## Data Availability

Original datasets of the current study and analyses generated are available in the BioStudies repository (https://wwwdevi.ebi.ac.uk/biostudies/Eu-ToxRisk/).
