# Supplementary material for: The sensitivity of the zebrafish embryo coiling assay for the detection of neurotoxicity by compounds with diverse modes of action
Source: Environ Sci Pollut Res Int. 2023 May 22;30(30):75281–99. doi: 10.1007/s11356-023-27662-2 (PMC10293418; doi:10.1007/s11356-023-27662-2)
Supplement: Supplementary file 1 — Supplementary file1 (DOCX 56 KB) [file 11356_2023_27662_MOESM1_ESM.docx]

**Supplementary Material 1.** Settings of the parameters for the assessment of all test compounds in the Danioscope Software (Version 1.1)

| **Compound** | **Activity**  **onset [%]** | **Activity**  **offset [%]** | **Minimum inter-**  **peak interval [ms]** | **Minimum peak**  **duration [ms]** |
| --- | --- | --- | --- | --- |
| Acrylamide | 2 | 0.5 | 100 | 0 |
| Carbaryl | 2 | 0.5 | 300 | 0 |
| Hexachlorophene | 2 | 0.5 | 300 | 0 |
| Ibuprofen | 2 | 0.5 | 100 | 0 |
| Rotenone | 2 | 0.5 | 300 | 0 |

**Supplementary Material 2.** Percentage of embryos analysed per concentration and time point that were viable for further statistical analysis

**Supplementary Material 3.** Video example of carbaryl exposed organisms at 35 hpf (4x recording speed). Red rings indicate individuals which were excluded from the 35 hpf analysis due to excessive movement

**The video is in the PowerPoint document due to the annotations**

**Supplementary Material 4.** p-Values for the coiling assay replicates noted as significant. At least 2 replicates had to give a level of significance. In the case of multiple replicates providing the same level of significance, the p-value was noted only once.

|  | **Mean burst duration** | | | **Mean burst count per minute** | | |
| --- | --- | --- | --- | --- | --- | --- |
|  | **Concentration** | **Age (h)** | ***p*-Value(s)** | **Concentration** | **Age (h)** | ***p*-Value(s)** |
| **Acryl­amide** | 1.3 mM | 45 | ≤ 0.05, ≤ 0.01 | 1.3 mM | 42 | ≤ 0.05 |
|  | 2.1 mM | 45 | ≤ 0.01, ≤ 0.001 |  | 43 | ≤ 0.05, ≤ 0.01 |
|  |  |  |  |  | 44 | ≤ 0.05, ≤ 0.01 |
|  |  |  |  | 2.1 mM | 38 | ≤ 0.05, ≤ 0.01 |
|  |  |  |  |  | 39 | ≤ 0.05, ≤ 0.01 |
|  |  |  |  |  | 40 | ≤ 0.05, ≤ 0.01 |
|  |  |  |  |  | 41 | ≤ 0.05 |
|  |  |  |  |  | 45 | ≤ 0.01 |
| **Carbaryl** | 37.3 µM | 24 | ≤ 0.05, ≤ 0.001 | 14.9 µM | 24 | ≤ 0.01, ≤ 0.0001 |
|  |  |  |  |  | 27 | ≤ 0.05 |
|  |  |  |  | 29.8 µM | 24 | ≤ 0.01, ≤ 0.001 |
|  |  |  |  |  | 26 | ≤ 0.01 |
|  |  |  |  |  | 27 | ≤ 0.05, ≤ 0.01 |
|  |  |  |  |  | 28 | ≤ 0.05 |
|  |  |  |  | 37.3 µM | 23 | ≤ 0.01 |
|  |  |  |  |  | 24 | ≤ 0.05, ≤ 0.01, ≤ 0.0001 |
| **Ibuprofen** | 48.5 µM | 30 | ≤ 0.05, ≤ 0.01 | 48.5 µM | 36 | ≤ 0.01, ≤ 0.001 |
|  | 145.4 µM | 24 | ≤ 0.05, ≤ 0.01 |  | 37 | ≤ 0.05, ≤ 0.01, ≤ 0.0001 |
|  |  | 30 | ≤ 0.01 |  | 38 | ≤ 0.05, ≤ 0.01 |
|  |  | 44 | ≤ 0.05, ≤ 0.0001 |  | 39 | ≤ 0.01 |
|  |  | 47 | ≤ 0.01 |  | 40 | ≤ 0.001, ≤ 0.0001 |
|  |  |  |  |  | 42 | ≤ 0.001, ≤ 0.0001 |
|  |  |  |  |  | 45 | ≤ 0.05, ≤ 0.01 |
|  |  |  |  |  | 47 | ≤ 0.05, ≤ 0.0001 |
|  |  |  |  | 145.4 µM | 24 | ≤ 0.05, ≤ 0.01, ≤ 0.0001 |
|  |  |  |  |  | 25 | ≤ 0.05, ≤ 0.001 |
|  |  |  |  |  | 26 | ≤ 0.01, ≤ 0.001 |
|  |  |  |  |  | 27 | ≤ 0.001 |
|  |  |  |  |  | 28 | ≤ 0.0001 |
|  |  |  |  |  | 29 | ≤ 0.01, ≤ 0.0001 |
|  |  |  |  |  | 30 | ≤ 0.05, ≤ 0.01, ≤ 0.0001 |
|  |  |  |  |  | 36 | ≤ 0.0001 |
|  |  |  |  |  | 38 | ≤ 0.001, ≤ 0.0001 |
|  |  |  |  |  | 39 | ≤ 0.0001 |
|  |  |  |  |  | 40 | ≤ 0.0001 |
|  |  |  |  |  | 41 | ≤ 0.0001 |
|  |  |  |  |  | 42 | ≤ 0.001, ≤ 0.0001 |
|  |  |  |  |  | 43 | ≤ 0.01, ≤ 0.0001 |
|  |  |  |  |  | 44 | ≤ 0.01, ≤ 0.0001 |
|  |  |  |  |  | 45 | ≤ 0.001, ≤ 0.0001 |
|  |  |  |  |  | 46 | ≤ 0.0001 |
|  |  |  |  |  | 47 | ≤ 0.0001 |
| **Rotenone** | 10.1 nM | 21 | ≤ 0.05 | 1.0 nM | 22 | ≤ 0.01, ≤ 0.0001 |
|  | 1.0 nM | 22 | ≤ 0.05, ≤ 0.001 |  |  |  |
