# Supplementary figures and images for: The sensitivity of the zebrafish embryo coiling assay for the detection of neurotoxicity by compounds with diverse modes of action
Source: Environ Sci Pollut Res Int. 2023 May 22;30(30):75281–99. doi: 10.1007/s11356-023-27662-2 (PMC10293418; doi:10.1007/s11356-023-27662-2)

## Slide 1
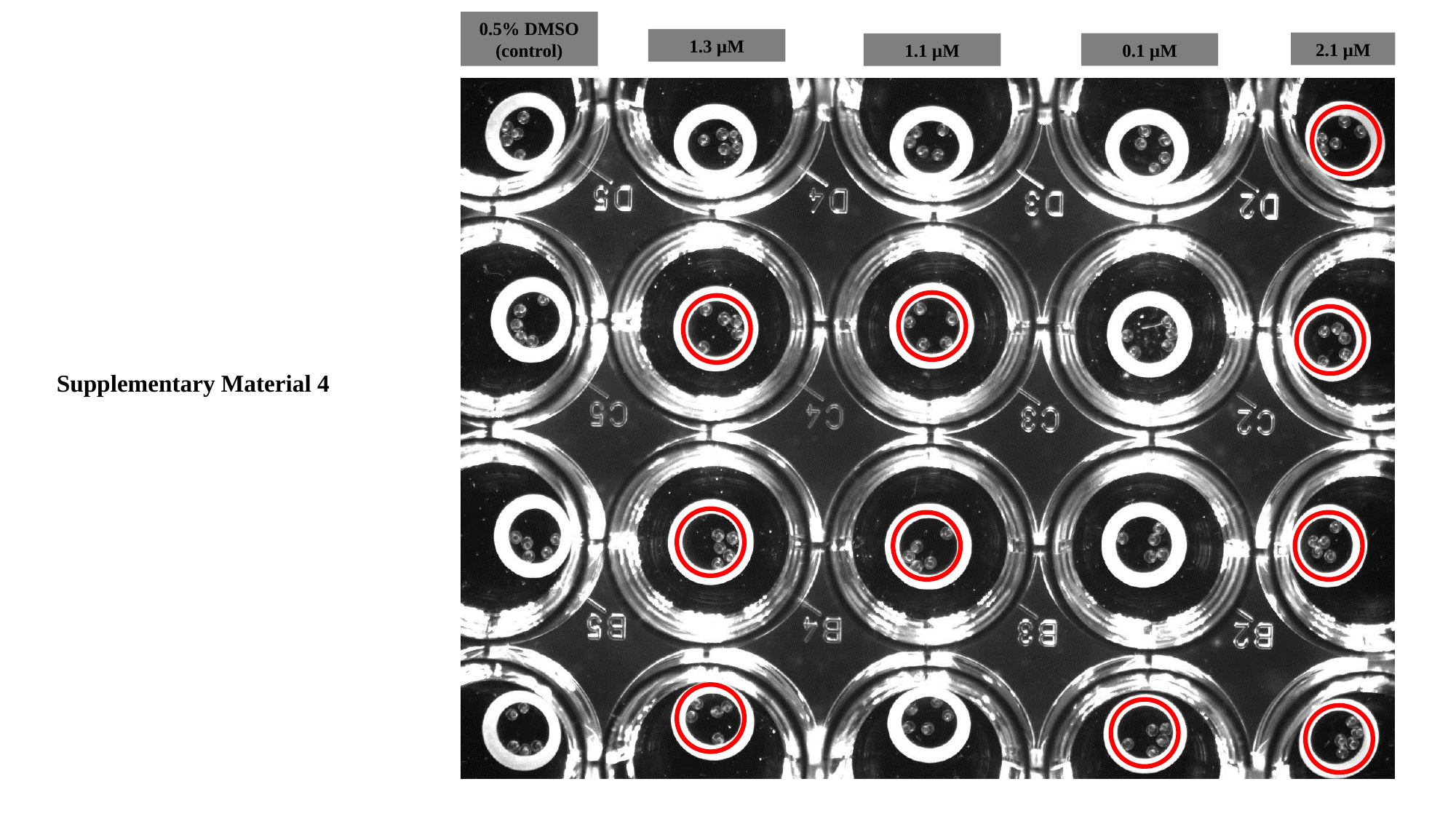

0.5% DMSO
(control)
1.3 µM
2.1 µM
0.1 µM
1.1 µM
Supplementary Material 4

Supplement: Supplementary file 2 — Supplementary file2 (PPTX 64231 KB) [file 11356_2023_27662_MOESM2_ESM.pptx]
